# Phosphorus Recovery by Adsorption from the Membrane Permeate of an Anaerobic Membrane Bioreactor Digesting Waste-Activated Sludge

**DOI:** 10.3390/membranes12010099

**Published:** 2022-01-17

**Authors:** Akira Hafuka, Katsuki Kimura

**Affiliations:** Division of Environmental Engineering, Graduate School of Engineering, Hokkaido University, North-13, West-8, Kita-ku, Sapporo 060-8628, Japan; ahafuka@eng.hokudai.ac.jp

**Keywords:** biogas, methane, sewage sludge, phosphorus release, zirconium, adsorbent, polytetrafluoroethylene, hollow fiber microfiltration, membrane fouling

## Abstract

The recovery of phosphorus (P) from waste activated sludge (WAS) is a promising approach for sustainable resource management. During the anaerobic digestion of WAS, orthophosphate is released, and this P species is favorable for adsorption recovery. In the present study, an anerobic membrane bioreactor (AnMBR) with a P-adsorption column was developed to generate biogas from WAS and to recover P from membrane permeate simultaneously. The effects of the hydraulic retention time (HRT) and solid retention time (SRT) of the AnMBR on P solubilization were investigated. As a result, the maximum P solubilization was 21% when the HRT and SRT were 45 days and 100 days, respectively. Orthophosphate in the membrane permeate was adsorbed and recovered using a mesoporous material called zirconium sulfate–surfactant micelle mesostructure (ZS) in the column. The adsorbed P could be desorbed from the ZS with a NaOH solution, and P was recovered as a concentrated solution by a factor of 25. When the HRT was 19 days, the biogas yield and biogas production rate were 0.26 L/g-VS_input_ and 0.123 L/L/d, respectively. The average methane content in the biogas was 80%. The developed membrane-based process may be effective for resource recovery from WAS.

## 1. Introduction

Phosphorus (P) is an essential nutrient for the normal growth of living organisms, and it is a valuable resource for agricultural and industrial use. However, existing phosphorus rock is limited and will be exhausted in 50–100 years [1]. Furthermore, phosphorus rock is unevenly distributed around the world [2]. In Japan, all phosphate rock is imported, and approximately 10% of the total amount of consumed P flows into the sewage system [3]. Phosphorus removal is often required in wastewater treatment plants (WWTPs) because the release of excess P into bodies of water can lead to eutrophication [4]. As a result of wastewater treatment, phosphorus is transferred and condensed into sludge [5]. Not only P removal, but also P recovery is needed in WWTPs for sustainable resource management [6].

The activated sludge process is the most widespread process in WWTPs, and it produces a large amount of waste-activated sludge (WAS) [7]. WAS is rich in P, and it also contains a large amount of organic matter (approximately 80% of the total solids) [8]. Therefore, energy can be recovered from WAS as methane-containing biogas by anaerobic digestion. Anaerobic digestion also achieves reductions in the sludge volume and the destruction of pathogens [9]. During the anaerobic digestion process, the phosphorus fractions in WAS are changed by the anaerobic conditions and microbial activity [10,11,12]. Specifically, poly-phosphate, which is a main P fraction in WAS, is released and hydrolyzed into orthophosphate.

During the anaerobic digestion of WAS with conventional continuously stirred tank reactors (CSTRs), a large reactor footprint is needed due to the sludge’s long retention time (~30 days). In addition, the quality of the effluent is generally poor because of the washout of the suspended solids (SS), including anaerobic microbes. To solve these problems, anaerobic membrane bioreactors (AnMBRs) have been applied to sludge digestion [13]. An AnMBR comprises a system that couples membrane filtration with anaerobic treatment. The distinctive feature of AnMBRs is the decoupling of the hydraulic retention time (HRT) and solid retention time (SRT). Anaerobic membrane bioreactors can shorten the HRT without simultaneously shortening the SRT, which reduces the footprint of the reactor [14]. In addition, anaerobic membrane bioreactors generate higher quality effluent than CSTRs because the membrane removes the SS. Undigested sludge and slow-growing microbes can be retained inside the digester due to solid–liquid separation by the membrane.

As described above, the orthophosphate concentration increases during anaerobic digestion of WAS, and its concentration is therefore high (~200 mg P/L) in the membrane permeate from the AnMBRs digesting WAS [15,16]. As orthophosphate is a favorable P species for its recovery, P recovery from the AnMBR permeate could be a promising approach for P recycling. However, studies focusing on P recovery from the AnMBR permeate are extremely limited [17,18]. Heronemus et al. have recently reported P recovery from the AnMBR permeate with chemical precipitation [17]. However, the P concentration in the membrane permeate was low (~4 mg P/L) in their study because AnMBR was used to treat sewage wastewater (not WAS). Therefore, a large amount of chemicals (aluminum chlorohydrate or FeCl_3_) was needed to achieve efficient P recovery in their study. Among the phosphorus-recovery methods, adsorption could be a suitable method for P recovery from the AnMBR permeate because of its high efficiency, simplicity, and ease of operation [19,20]. The orthophosphate ion is a favorable P species for adsorption, and most adsorbents are reusable. Furthermore, the AnMBR permeate does not contain SS, which is beneficial for the adsorption process because it prevents the clogging of the adsorption column and enables the easy separation and reuse of the adsorbent. Zhao et al. have recently reported P recovery from the P-enriched brine of the integrated AnMBR-reverse osmosis ion exchange process with nano-sorbents [18]. However, municipal wastewater was used as raw wastewater in their study, and the P concentration in the permeate was therefore low, at around 30 mg P/L. Therefore, phosphorus recovery from the membrane permeate from AnMBRs treating WAS has not been demonstrated.

We previously developed a novel adsorbent for orthophosphate known as the zirconium sulfate–surfactant micelle mesostructure (ZS) [21,22]. The adsorbent can remove orthophosphate ions from water through an ion exchange process with high selectivity and a high adsorption capacity (114 mg P/g-ZS). In this study, to recover P and to generate biogas from WAS simultaneously, an AnMBR with a P-adsorption column was developed. The column was filled with the ZS and the reactor was continuously operated. We hypothesized that the HRT and SRT of the AnMBR would affect P solubilization. In addition to the P solubilization, the anaerobic digestion, the P-adsorption column, and the membrane unit performance were also evaluated.

## 2. Materials and Methods

### 2.1. Reactor Setup and Operating Conditions

**Figure 1:**
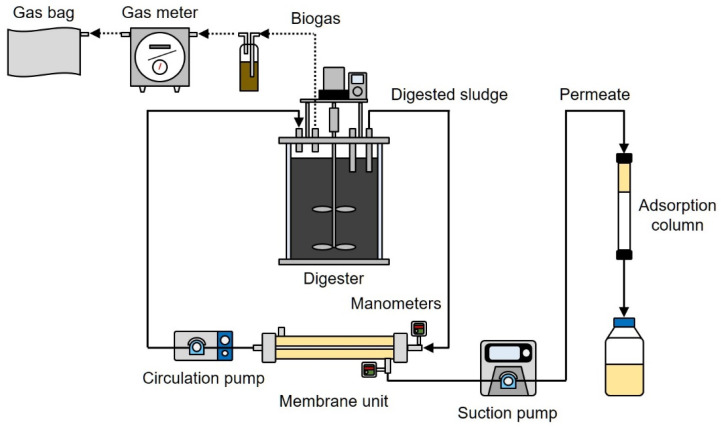
Schematic diagram of the reactor.

A schematic diagram of the AnMBR experimental apparatus is shown in Figure 1. The apparatus comprised a digester, an external membrane unit, and a phosphate adsorption column. A digester with a working volume of 6.0 L was continuously mixed with a stirrer at 30 rpm. The temperature was held constant at 37 °C (i.e., the mesophilic condition) using a water jacket. The external membrane unit had a working volume of 0.23 L, and the unit contained a polytetrafluoroethylene hollow fiber microfiltration (MF) membrane (Poreflon, Sumitomo Electric Industries, Ltd., Osaka, Japan). The total effective area of the membrane was 0.0048 m^2^, the inner diameter of a membrane fiber was 5 mm, and the pore size of the membrane was 0.45 μm. The digester was fed with seed sludge (6.0 L) obtained from a full-scale mesophilic digester in a sewage treatment plant. The concentrations of total solids (TS) and volatile solids (VS) in the seed sludge were 13.1 and 8.9 g/L, respectively. The WAS fed into the AnMBR was obtained from another full-scale sewage treatment plant using a conventional activated sludge process. The raw WAS was screened through a 1 mm mesh screen, and then the supernatant was removed to thicken the sludge. The AnMBR was operated in semi-batch mode. Sludge feeding (0.70–1.05 L) and sludge withdrawal (0.42–0.70 L) were performed manually once or twice per week. The digested sludge was continuously circulated using a peristaltic pump, and the membrane filtration of the digested sludge was performed at a constant cross-flow velocity of 0.5 m/s. The digested sludge was continuously filtered through the hollow fiber membrane (i.e., inside-out filtration), and the membrane permeate was introduced into the glass column filled with phosphate adsorbent. The inner diameter and length of the column were 15 and 300 mm, respectively. The column was filled with 15 g of ZS (bed volume of 9 mL), which was prepared using a previously described method [21,22]. The membrane permeate was continuously passed through the column from top to bottom, and the column permeate was collected in a sampling bottle. The membrane flux was kept constant by controlling the filtrate flow rate with a peristaltic pump. The pressure inside and outside of the membrane was measured daily using manometers, and the transmembrane pressure (TMP) values were calculated from these data. The volume of the biogas produced in the digester was measured using a wet gas meter, and the biogas was stored in an aluminum gas bag. The HRT and SRT of the reactor were controlled by changing the filtrate flow and sludge withdrawal according to

(1) $$\mathrm{HRT(d)}=\frac{Volume\;of\;the\;reactor\;\left(L\right)}{Input\;of\;WAS\;\left(L/d\right)}$$

(2) $$\mathrm{SRT(d)}=\frac{Volume\;of\;the\;reactor\;\left(L\right)}{Withdrawal\;of\;digested\;sludge\;\left(L/d\right)}$$

The whole experiment was performed for 166 days, and it was divided into four separate phases, each involving distinct operational conditions (Table 1). During phase 1 (operation days 1–78), both the HRT and the SRT were set to 60 days (i.e., CSTR mode without using the membrane unit). Phase 1 was considered to be the acclimatization period. The operation of the AnMBR began in phase 2 (operation days 78–110), during which the HRT and SRT were set to 65 and 100 days, respectively. The HRT was shortened to 45 days while maintaining the SRT during phase 3 (operation days 110–138). For the final phase of the experiment, phase 4 (operation days 138–166), the HRT was further shortened to 19 days while maintaining the SRT.

### 2.2. Analytical Methods for Sludge and Membrane Permeate

Selected physical and chemical properties of the WAS, digested sludge, and membrane permeate were analyzed to characterize them. Unless otherwise stated, all chemical reagents were analytical grade and were purchased from Sigma-Aldrich Japan (Tokyo, Japan) or FUJIFILM Wako Pure Chemical Corporation (Osaka, Japan). The pH and oxidation reduction potential (ORP) of the samples were measured using a pH/ORP meter (D-73, Horiba, Ltd., Kyoto, Japan). The concentrations of the total solids (TS) and volatile solids (VS) were determined according to standard methods [23]. The chemical oxygen demand with potassium dichromate (COD_Cr_) and total phosphorus (T-P) concentrations were measured according to Hach methods (methods 8000 and 10127, respectively) using a spectrophotometer (DR 3900, Hach Co., Loveland, CO, USA) after appropriate sample dilution [24]. The ammonium nitrogen (NH_4_-N) and orthophosphate (PO_4_-P) concentrations were also determined following Hach methods (methods 10031 and 8114, respectively) [24]. Before determining the NH_4_-N and PO_4_-P concentrations, the sludge was diluted and centrifuged, and the supernatant was filtered through a 0.45-μm filter (25HP045AN, Toyo Roshi Kaisya, Ltd., Tokyo, Japan). The alkalinity of the digested sludge was determined using standard methods [23]. The solubilization ratio from T-P to PO_4_-P was calculated by

(3) $$Phosphorus\;solubilization\;\left(\%\right)=\frac{Increased\;mass\;of\;PO_{4}\text{-}P}{Supplied\;mass\;of\;T\text{-}P}\times 100$$

The efficiency of chemical oxygen demand (COD) rejection by the membrane was calculated by

(4) $$\mathrm{COD}\;rejection\;efficiency\;of\;the\;membrane\;\left(\%\right)=\frac{{\mathrm{COD}}_{digested\_sludge}-{\mathrm{COD}}_{permeate}}{{\mathrm{COD}}_{digested\_sludge}}$$

where COD*_digested_sludge_* (i.e., the total COD) and COD*_permeate_* (i.e., the soluble COD) are the COD concentrations of the digested sludge and membrane permeate (mg/L), respectively.

The methane and carbon dioxide contents of the biogas were determined with a gas chromatograph (GC-14B, Shimadzu Corporation, Kyoto, Japan) equipped with a thermal conductivity detector and a 6.0 m × 3.0 mm stainless steel-packed column (Shincarbon St, Shinwa Chemical Industries, Ltd., Kyoto, Japan). The particle-size distribution of ZS was determined using a nanoparticle size analyzer (SALD-7100, SHIMADZU Corp., Kyoto, Japan).

### 2.3. Phosphate Desorption from the Adsorbent

Phosphorus was desorbed from the adsorbent after phase 2 to recover the P. The adsorbent was transferred from the column to a polypropylene bottle. During the desorption process, 0.3 M NaOH (50 mL) was added to the bottle, and the mixture was stirred for 1 h. After stirring, the mixture was centrifuged at 5000 rpm for 10 min, and the supernatant was removed. The orthophosphate concentration in the supernatant was determined using the Hach method. The amount of desorbed phosphate was determined based on the amount of phosphate in the supernatant. The separated adsorbent was washed with ultrapure water, and the regenerated adsorbent was re-introduced into the column. Phosphate desorption was performed again after phase 4 by following the same procedure described above using 0.3 M NaOH (300 mL).

### 2.4. Ex Situ Membrane Cleaning

Ex situ cleaning of the membrane was performed after the reactor had been operating for 166 days to investigate the contribution of reversible and irreversible membrane fouling. The membrane unit was removed from the AnMBR and the inside of the hollow fiber membrane was washed with tap water to physically remove the cake layer. The membrane was soaked in a NaOH–NaClO aqueous solution (10 g/L NaOH and 1000 mg/L NaClO) for 24 h to remove the remaining foulants. Following cleaning with tap water and chemical solution, the filtration resistance of the membrane was determined by filtering tap water at a pressure of 70 kPa. The filtration resistance values before and after cleaning were compared to the filtration resistance of the pristine membrane, which was measured before reactor operation. The filtration resistance of the membrane was determined by

(5) $$R=\frac{\mathrm{TMP}\left(\mathrm{Pa}\right)}{\mu \;\left(\mathrm{Pa}\cdot s\right)\times J\;\left(m^{3}/\left(m^{2}\cdot s\right)\right)}$$

where *R* is the filtration resistance, *μ* is the viscosity of water, and *J* is the permeate flux.

## 3. Results and Discussion

### 3.1. Phosphorus Adsorption and Recovery

The average TS and VS concentrations of the WAS were 9.2 and 7.3 g/L, respectively (Table 2). The VS/TS ratio remained stable at approximately 79%, and the average total COD (T-COD) concentration in the WAS reached 12.5 g/L. The average T-P and PO_4_-P concentrations were 208 and 14.0 mg-P/L, respectively. This suggested that PO_4_-P was a minor P fraction of the WAS, and it accounted for approximately 7% of the T-P concentration in the WAS. The low contribution of PO_4_-P toward T-P in WAS has also been reported in a previous study [25].

**Figure 2:**
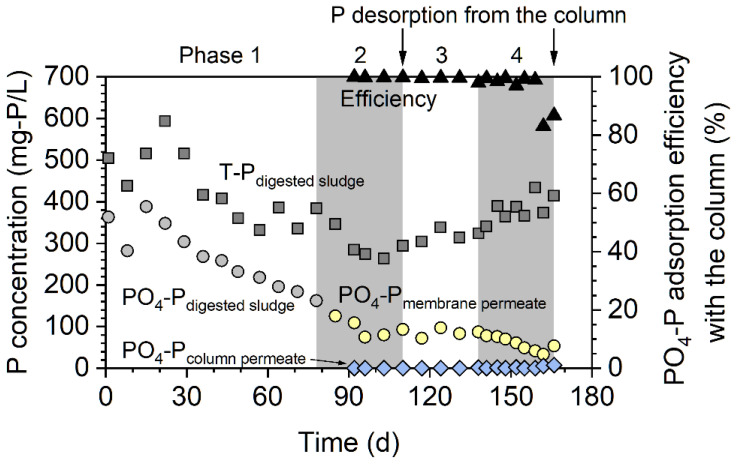
Total phosphorus and orthophosphate concentrations as well as the adsorption efficiency of orthophosphate to the column.

The digester was operated in the CSTR mode of operation during phase 1. Both the HRT and SRT were set to 60 days. During this phase, the T-P and PO_4_-P concentrations of the digested sludge gradually decreased due to sludge withdrawal (Figure 2). In phase 1, the average PO_4_-P to T-P ratio in the digested sludge was 61%, which was much higher than that in the WAS (i.e., 7%). This indicated that P solubilization occurred during anaerobic digestion. Considering the mass balance of P, P solubilization was estimated to be 22% in phase 1. This means that 22% of the P supplied to the digester (i.e., the sum of suspended P, poly-P, and dissolved organic P) was transformed to PO_4_-P under anaerobic conditions. The released PO_4_-P was estimated to be 6.2 mg P/g VS in this study. This value was higher than the previously reported value (0.59 mg P/g VS) [26].

From phase 2, the AnMBR mode of operation was started, and continuous P adsorption from the membrane permeate was performed using the column. From phase 2 to phase 4, the HRT was shortened from 65 to 19 days step by step while keeping the SRT fixed at 100 days (see Table 1). From the middle of phase 2, the T-P concentration gradually increased because the membrane filtration retained the digested sludge with prolonged SRT and increased its concentration. In contrast, the PO_4_-P concentration gradually decreased, especially during phase 4. Therefore, the PO_4_-P/T-P ratio decreased during AnMBR mode operation. At the end of phase 4, PO_4_-P only accounted for 10% of the T-P. Due to membrane filtration, PO_4_-P passes into the membrane and inert suspended phosphorus might accumulate inside the digester. In phase 2, phosphorus solubilization did not occur, and the phosphorus solubilization ratio was −3%. Although the SRT was prolonged to 100 days in phase 2, P solubilization was not accelerated in the AnMBR mode. Phosphorus solubilization occurred again in phase 3, and the phosphorus solubilization ratios were 21% and 15% in phases 3 and 4, respectively. These values were comparable to that in phase 1 (22%). This result suggested that the HRT has a great impact on P solubilization in the digester when the SRT was kept constant in the AnMBR mode.

**Figure 3:**
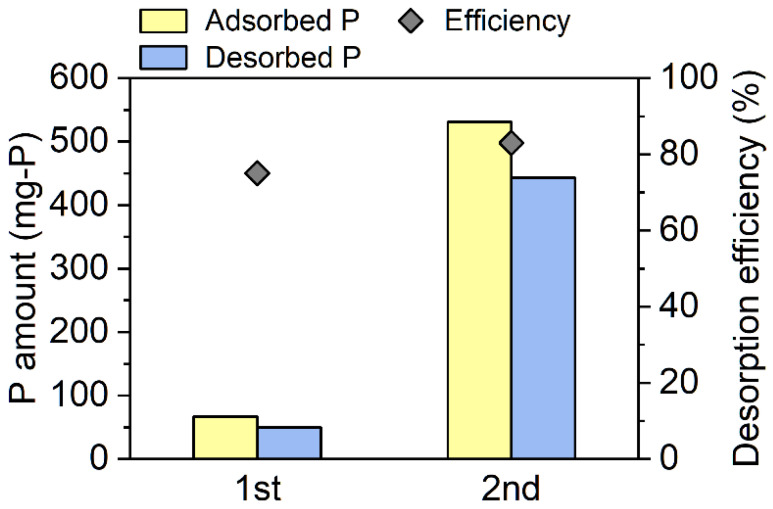
Amounts of phosphorus adsorbed on the adsorbent in the column during phase 2 and phases 3–4 as well as the amounts of phosphorus desorbed from the adsorbent. The diamonds indicate the phosphorus desorption efficiency.

Measurement using the nanoparticle size analyzer revealed that the median particle size of the ZS was 31 μm (see Figure S1 in Supplementary Materials). The P-adsorption column showed high adsorption efficiency for PO_4_-P, and it was above 99% during phase 2. In this phase, the PO_4_-P concentrations in the column permeate were below 0.2 mg-P/L. After phase 2, P desorption from the adsorbent was conducted, and the regenerated adsorbent was re-introduced into the glass column. As a result, the column maintained a high adsorption efficiency for PO_4_-P (>98%), which means that the adsorbent could be used repeatedly. A breakthrough point appeared on day 162 during phase 4, and the adsorption efficiency decreased to 83%. The second P desorption was conducted after phase 4. The amount of P adsorbed during phase 2 was 67 mg P (Figure 3). In the first desorption experiment, 50 mg P was desorbed, and the desorption efficiency reached 75%. The amount of P adsorbed during phases 3 and 4 (531 mg P) was larger than that adsorbed during phase 2 (67 mg P) due to the long-term operation of the adsorption column. The amount of P that was desorbed was 443 mg, and the desorption efficiency was 83%. During the second adsorption, 8.26 L of membrane permeate passed through the column (i.e., 918 bed volumes). The adsorption capacity of the ZS was calculated to be 35.4 mg-P/g-ZS, which was higher than those of other P adsorbents [27,28]. However, the adsorption capacity of the ZS was lower than that in the synthetic P solution (114 mg-P/g-ZS) [22]. A previous study revealed that the bicarbonate ion is a competing anion for the adsorption of the orthophosphate ion on ZS [22]. Therefore, the bicarbonate ion in the membrane might have interfered with P adsorption and may have decreased the adsorption capacity in the present study. Overall, a total of 8.82 L membrane permeate passed through the column (i.e., 980 bed volumes) during phases 2–4. Phosphorus was desorbed from the column with 350 mL of NaOH solution; therefore, P was concentrated 25 times and was recovered as a concentrated solution. This concentration factor was higher than that obtained in a previous study [18].

### 3.2. Performance of Digestion and Membrane Separation

The digestion performance of the reactor during each phase is summarized in Table 3. The decrease in the HRT in the AnMBR from 65 to 19 days led to an increase in the organic loading rate (OLR) from 0.12 to 0.47 g-VS/L/day, which further enhanced the biogas production rate from 0.057 to 0.123 L/L/day. The methane content in the biogas was stable at around 80%. Although the biogas production rate increased, the biogas yield gradually decreased from 0.35 to 0.26 L/g-VS_input_ during reactor operation. This could be because soluble organic matter, such as volatile fatty acids, might pass through the membrane in the AnMBR operation mode, which decreases the biogas yield [29].

**Figure 4:**
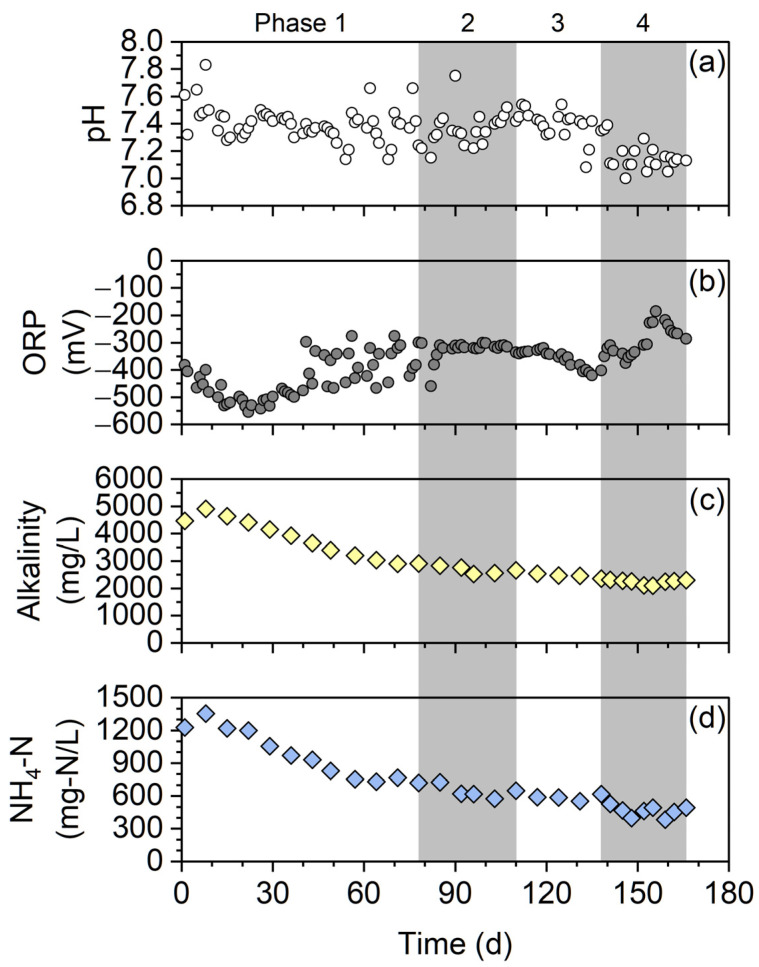
(**a**) pH, (**b**) ORP, (**c**) alkalinity, and (**d**) NH_4_-N concentration in the digestor.

The pH fluctuated within the range 7.0–7.8, but it did not greatly deviate from the optimum pH range of 7.0–7.4 for anaerobic digestion (Figure 4a) [9]. However, the ORP was slightly higher in the latter part of phase 4 (above −300 mV, Figure 4b). This might be caused by the shortened HRT (i.e., 19 days) in phase 4. The alkalinity and NH_4_-N concentration gradually decreased during reactor operation (Figure 4c,d). The NH_4_-N concentration was below 1500 mg-N/L during operation and remained below the inhibition level [30].

**Figure 5:**
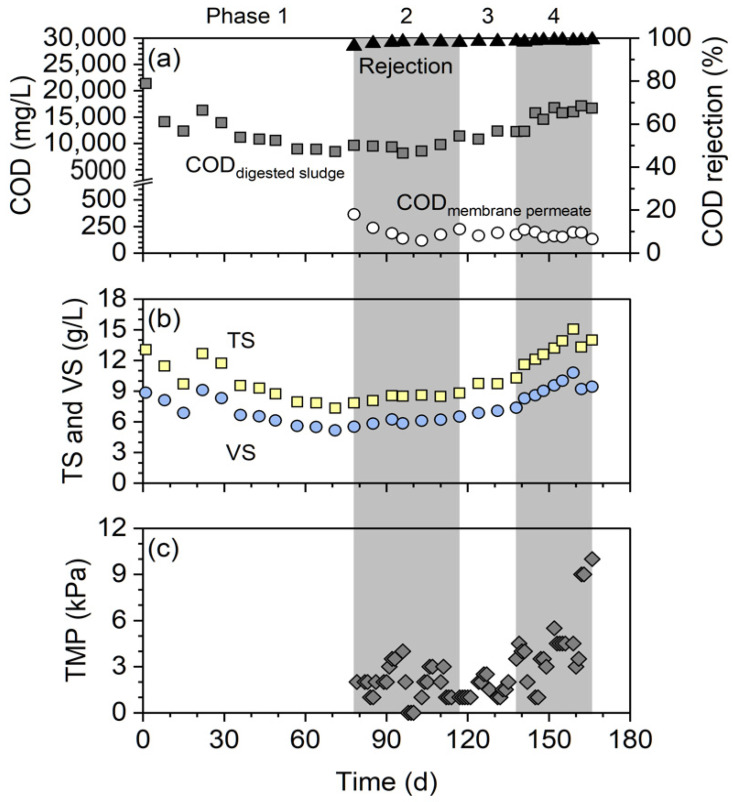
(**a**) COD concentrations in the digested sludge and membrane permeate as well as the COD rejection efficiency by the membrane. (**b**) TS and VS concentrations in the digested sludge. (**c**) TMP of the membrane unit.

The COD concentration in the digested sludge (i.e., T-COD) was in the range 8200–21,400 mg/L, and it gradually increased from 9600 to 16,700 mg/L during phases 2–4, while membrane filtration continued (Figure 5a). During phases 2–4, the COD concentration in the membrane permeate (i.e., the soluble COD) stabilized at 190 mg/L (Figure 5a). Overall, the efficiency of COD rejection by the membrane was greater than 96%. This is attributed to the membrane’s highly efficient rejection of suspended COD. The same trend was observed for the TS and VS concentrations (Figure 5b). Solid–liquid separation by the membrane increased the concentrations of T-COD, TS, and VS during phases 2–4. The membrane thickened the digested sludge and helped retain it inside the digester, which led to a higher content of microbial biomass. This originated from a distinctive feature of AnMBRs, that is, the decoupling of the HRT and SRT. AnMBRs can prolong the SRT without simultaneously extending the HRT.

Although membrane fouling is inevitable in membrane-based treatment processes, severe membrane fouling was not observed in this study (Figure 5c). The TMP was stable at below 10 kPa for 89 days of continuous filtration without any membrane cleaning (phases 2–4). There are two reasons for the successful mitigation of membrane fouling. The first reason is that the reactor configuration incorporated external cross-flow. There are two types of AnMBR configurations: external cross-flow and submerged configurations [13]. In external cross-flow configurations, the membrane unit is outside the digester, whereas in the submerged configurations, the membranes are immersed in the digester or external sludge tank. The high shear force on the membranes in a cross-flow configuration can better control membrane fouling. In addition, the use of an external membrane unit outside the digester can provide the ease of membrane maintenance because the head space of the digester cannot be frequently opened to maintain anaerobic conditions. Although the cross-flow velocity of 0.5 m/s in the present study required additional energy (to power the cross-flow pumps), it was proven that to mitigate membrane fouling, energy can be recovered from the anaerobic digestion system in the form of biogas. The second reason is that the relatively low filtration flux (i.e., 2.08 LMH) effectively prevents a drastic increase in the TMP. AnMBRs that are used to treat WAS have relatively long HRTs compared to the aerobic or anaerobic membrane bioreactors that are used to treat low-strength wastewater. Therefore, a relatively low flux is often sufficient for WAS digestion in AnMBRs, which can mitigate membrane fouling [15,16].

**Figure 6:**
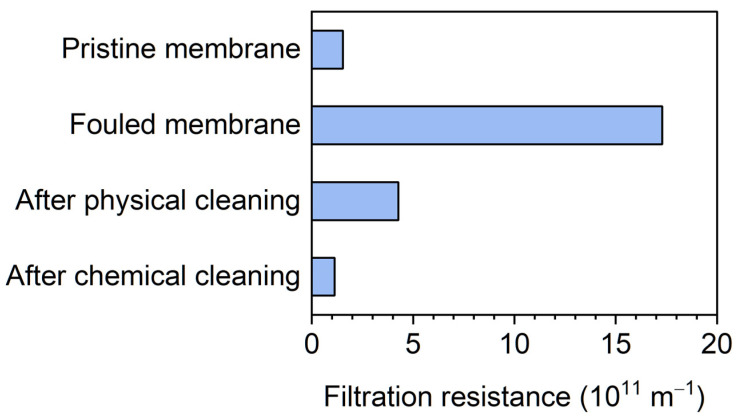
Filtration resistance values of the pristine membrane, fouled membrane, and cleaned membrane. Physical and chemical cleaning were sequentially performed.

Membrane fouling can be mainly classified as reversible and irreversible fouling [31]. Reversible fouling is mainly caused by cake-layer formation (deposition of biosolids), and this fouling can be removed by physical cleaning. In contrast, irreversible fouling cannot be removed by physical cleaning, and it is also called physically irreversible fouling. Irreversible fouling can be removed by chemical cleaning. Ex situ membrane cleaning was performed after the reactor had been operating for 166 days to identify which type of fouling was dominant in the present study (Figure 6). The filtration resistance of the pristine membrane was 1.5 × 10^11^ m^−1^. After the membrane unit had been operating for 89 days, the resistance increased to 17.3 × 10^11^ m^−1^ due to membrane fouling. Hydraulic physical cleaning removed the membrane foulants, and the resistance decreased to 4.3 × 10^11^ m^−1^. This means that the physical cleaning was effective, and it recovered 82% of the water permeability of the membrane. Subsequent chemical cleaning with a NaOH–NaClO solution was performed to remove physically irreversible fouling. This cleaning further decreased the resistance to 1.1 × 10^11^ m^−1^, which was comparable to that of the pristine membrane. Therefore, physically irreversible fouling was successfully removed by chemical cleaning. Overall, physically reversible fouling was dominant in the AnMBR. The combination of physical and chemical cleaning successfully recovered the water permeability of the membrane.

## 4. Conclusions

An AnMBR with a P-adsorption column has been developed to generate biogas and to recover P from WAS simultaneously. Membrane separation of the digested sludge provided an orthophosphate-rich membrane permeate, which was beneficial for P recovery. The maximum P solubilization was 21% in the AnMBR. The HRT had a great impact on P solubilization in the digester. This low P solubilization is not attractive, and therefore, the future research is needed to improve it. During the 89 days in which the AnMBR was operating, the adsorption column maintained a high adsorption efficiency (>98%) for orthophosphate in the membrane permeate. The phosphorus was desorbed from the adsorbent with high levels of efficiency using a NaOH solution, and P was concentrated 25 times. Physically reversible fouling was dominant in the membrane, and subsequent chemical cleaning recovered the water permeability of the membrane.

## Figures and Tables

**Table 1:** Operational conditions of each phase of reactor operation.

Phase	Operational Mode	HRT (d)	SRT (d)	Filtration Flux (LMH)	Column Flow Rate (mL/h)
1	CSTR	60	60	-	-
2	AnMBR	65	100	0.33	1.98
3	AnMBR	45	100	0.79	4.12
4	AnMBR	19	100	2.08	9.11

**Table 2:** Characteristics of the raw WAS (*n* = 28).

Parameters (Unit)	Values ± Standard Deviations
TS (g/L)	9.2 ± 2.8
VS (g/L)	7.3 ± 2.2
VS/TS (%)	79 ± 3
T-COD (g/L)	12.5 ± 3.8
NH4-N (mg-N/L)	13.5 ± 10.8
T-P (mg-P/L)	208 ± 64
PO4-P (mg-P/L)	14.0 ± 9.2

**Table 3:** WAS digestion performance of the reactor during each phase.

Phase	Organic Loading Rate (g-VS/L/d)	Biogas Yield (L/g-VS_input_)	Biogas Production Rate (L/L/d)	CH_4_ Content (%)
1	0.12	0.35	0.043	- ^1^
2	0.17	0.34	0.057	79.7 ± 2.4
3	0.25	0.28	0.071	80.8 ± 2.1
4	0.47	0.26	0.123	81.0 ± 3.8

^1^ A biogas sample was not recovered.

## Data Availability

Not applicable.

## Supplementary Materials

The following are available online at https://www.mdpi.com/article/10.3390/membranes12010099/s1, Figure S1: Particle size distribution of ZS.
