# Supplementary material for: Phosphorus Recovery by Adsorption from the Membrane Permeate of an Anaerobic Membrane Bioreactor Digesting Waste-Activated Sludge
Source: Membranes (Basel). 2022 Jan 17;12(1):99. doi: 10.3390/membranes12010099 (PMC8778099; doi:10.3390/membranes12010099)
Supplement: Supplementary file 1 [file membranes-12-00099-s001.zip › membranes-1526158-supplementary.pdf]

membranes

Article

Phosphorus Recovery by Adsorption from the Membrane Permeate of an Anaerobic Membrane Bioreactor Digesting Waste-Activated Sludge

Akira Hafuka and Katsuki Kimura *

Division of Environmental Engineering, Graduate School of Engineering, Hokkaido University, North-13, West-8, Kita-ku, Sapporo 060-8628, Japan; ahafuka@eng.hokudai.ac.jp
* Correspondence: kkatsu@eng.hokudai.ac.jp

Supplementary material

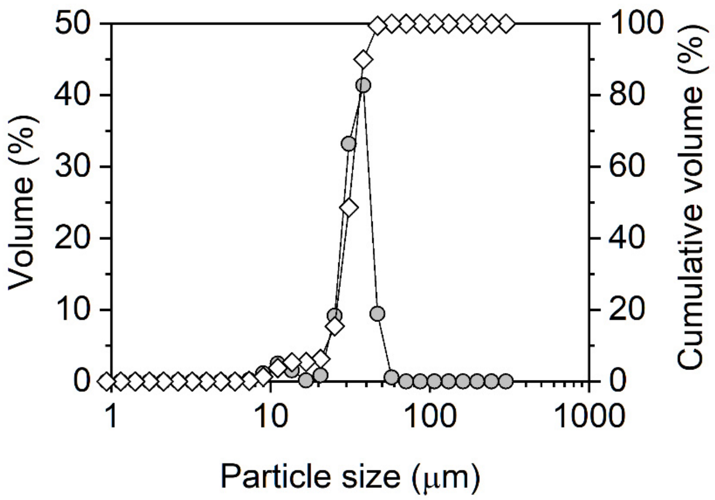

Figure S1. Particle size distribution of ZS.

Citation: Hafuka, A.; Kimura, K. Phosphorus Recovery by Adsorption from the Membrane Permeate of an Anaerobic Membrane Bioreactor Digesting Waste-Activated Sludge. *Membranes* **2022**, *12*, 99. https://doi.org/10.3390/membranes12010099

Academic Editor: Jia Wei Chew

Received: 10 December 2021
Accepted: 13 January 2022
Published: 17 January 2022

Publisher's Note: MDPI stays neutral with regard to jurisdictional claims in published maps and institutional affiliations.

Copyright: © 2022 by the authors. Submitted for possible open access publication under the terms and conditions of the Creative Commons Attribution (CC BY) license (https://creativecommons.org/licenses/by/4.0/).
